# An Automatic Field Plot Extraction Method From Aerial Orthomosaic Images

**DOI:** 10.3389/fpls.2019.00683

**Published:** 2019-05-29

**Authors:** Zohaib Khan, Stanley J. Miklavcic

**Affiliations:** School of Information Technology and Mathematical Sciences, Phenomics and Bioinformatics Research Centre, University of South Australia, Adelaide, SA, Australia

**Keywords:** plot extraction, precision phenotyping, aerial image analysis, remote sensing, unmanned aerial systems

## Abstract

Unmanned aerial vehicles have an immense capacity for remote imaging of plants in agronomic field research trials. Traits extracted from the plots can explain development of the plants coverage, growth, flowering status, and related phenomenon. An important prerequisite step to obtain such information is to find the exact position of plots to extract them from an orthomosaic image. Extraction of plots using tools which assume a uniform spacing is often erroneous because the plots may neither be perfectly aligned nor equally distributed in a field. A novel approach is proposed which uses image-based optimization algorithm to find the alignment of plots. The method begins with a uniformly spaced grid of plots which is iteratively aligned with regions of high vegetation index, i.e., the underlying plots. The approach is validated and tested on two different orthomosaic images of fields containing wheat plots with simulated and real alignment problems, respectively. The result of alignment is compared to manually located ground truth position of plots and the errors are quantitatively analyzed. The effectiveness of the proposed method is confirmed in accurately estimating the phenotypic trait of canopy coverage compared to the common methods of extraction from uniform grids or trimmed grids. The software developed in this study is available from SourceForge, https://sourceforge.net/projects/phenalysis/.

## 1. Introduction

The United Nations estimates world population to reach 9.8 billion in 2050 (United Nations, 2017). To meet the growing demand of food with limited resources, production needs to increase by 70%, most of which relates to the cereals (Alexandratos and Bruinsma, 2012). Efforts to identify cultivation varieties which can perform under extreme climatic conditions have been accelerated by breeding programs that aim to bring resilience to drought, heat and salinity in a plant species (Wang et al., 2003; Tricker et al., 2018). Breeding is carried out through recurring cycles of “crossing,” “selection,” and “elimination” of varieties grown in different environmental conditions over several generations. In general, hundreds of varieties may be sown in field plots (also known as micro-plots or research plots) from which a few superior varieties are selected for successive evaluation cycles. Between sowing and harvest, breeders scout the field going from plot to plot and visually assign scores based on qualitative and quantitative traits of plants (e.g., height, vigor, flowering, leaf area, growth stage) for ranking at multiple stages of development. A major drawback is that manual assessment is labor intensive, subjective, and prone to human error.

Agricultural machinery has brought significant automation to farming activities. Today, mechanized seeders can sow varieties at a prescribed rate in uniformly spaced single row or multi-row plots in a field (Unruh, 2015). Subsequently, combined harvesters separately thresh each plot in sequence to independently record the varietal yield from each plot (Argetsinger et al., 2010). Driven by automation, sensor-based phenotyping platforms are emerging as an alternative to manual field phenotyping. Unmanned aerial vehicle (UAV), in particular, are now being used to acquires images of field plots in an efficient and non-invasive manner. Using photogrammetry software, aerial images are stitched together to generate an orthomosaic image which gives a holistic view of all field plots.

Delineation of field plots from the orthomosaic image is a preliminary step for plot-level analysis of attributes. The number of seedlings emerging from the seeds planted per plot is important for early intervention and management (Sankaran et al., 2015). Estimate of plot coverage is widely considered as a performance trait of developing plants (Duan et al., 2017). The number of flowers or fruits per plot is an indirect estimator of prospective yield (Xu et al., 2018). In general, many biophysical properties can be associated by correlation to vegetation indices derived from the plots (Lelong et al., 2008; Di Gennaro et al., 2017). The accuracy of such tasks is dependent on accurate delineation of field plots from an orthomosaic image. However, several issues hinder the extraction using primitive information such as plot size and spacing. Practically, the following issue(s) may arise:

1. Plots sown away due to their placement along the track of mechanical seeder.
2. Plots appear in ambiguous location due to partial emergence of seedlings.
3. Plots not aligned with the sown position due to geo-referencing error of the orthomosaic.

Methods to extract plots from aerial images range in manual, semi-automatic, or automatic. The manual approach is to mark polygonal regions, which can be particulary suitable for arbitrary shaped canopies (Virlet et al., 2014). However, the process of marking of position or sequence of plots can be tedious as well as erroneous. An automatic approach to delineate the extents of a plot is by classifying image pixels into “plant” and “non-plant” categories (Recio et al., 2013; Haghighattalab et al., 2016). Then, the minimum bounding box around each isolated cluster of plant pixels is regarded as the plot boundary. However, such methods assume ideal segregation between plots, failing which multiple plots may be seen as a single plot.

**Figure 1:**
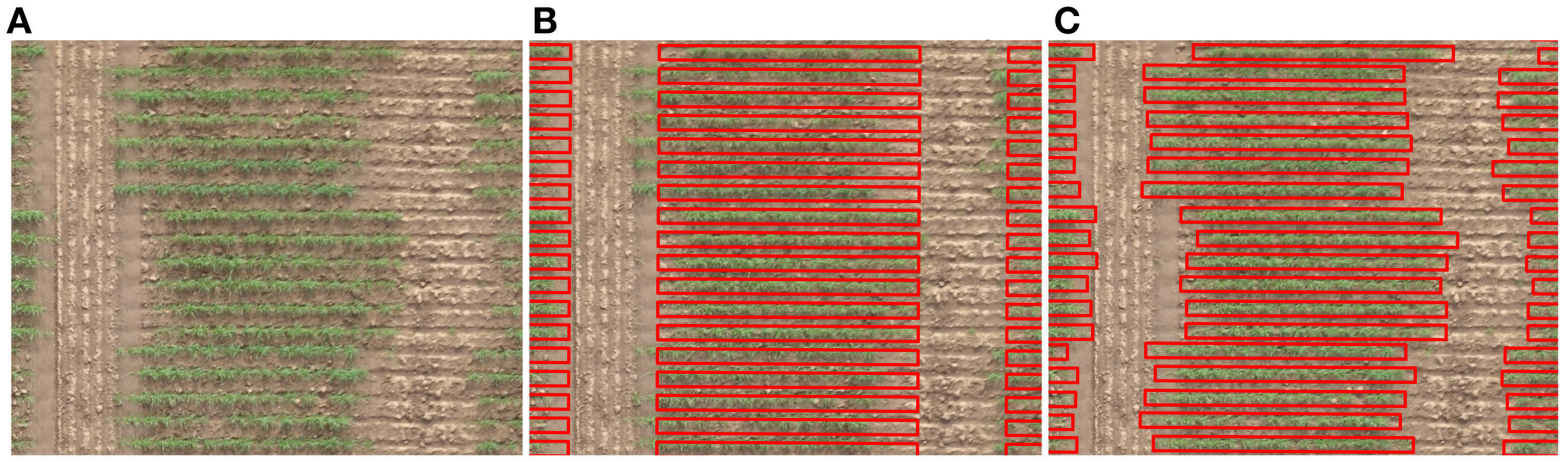
Aligning a grid of cells with an aerial view of an agronomic field trial. **(A)** An irregularly spaced range of plots planted in single rows. **(B)** A regularly spaced range of cells in gross misalignment with plots. **(C)** The ground truth positioning of cells in alignment with actual plot location.

A semi-automatic approach is to mark the extent of a trial such that it can be split into equally sized plots (Deery et al., 2016; Duan et al., 2017). The user manually marks the bounding corners of a trial which is automatically divided into a grid of cells based on the number of rows and columns, and reduced margin to remove the alleyway. However, if the plots are non-uniformly spaced, delineation will be inaccurate because a uniformly spaced grid will not align with such plots as shown in Figure 1. A common workaround is to limit the plot bounds by clipping them to a smaller, central portion for analysis. As a consequence, the user is deprived of the full distribution of attributes obtainable from whole plots.

Precise location of plots is partially dependent on accurate geo-referencing of an orthomosaic. Theoretically, if a mosaic is perfectly geo-registered, plots can be extracted based on the map coordinates prescribed in the sowing plan (Hearst and Cherkauer, 2015). Ground control point (GCP) objects are commonly used to geo-reference imagery and enable plot extraction with sufficient accuracy. However, use of GCP only mitigates the global image referencing error. It is important to note that recovery of irregular space variation between plots is not addressable through such methods.

In this paper, we present a semi-automatic method for accurate extraction of plots from an aerial orthomosaic image. The method assumes that a cellular grid based on the number of plots in rows and columns has been coarsely laid over the image. This assumption is practically feasible using information which is readily available from a trial design as modern sowing machines can make use of GPS based plot locations. The position of grid cells is then automatically optimized such that each cell accurately aligns with the underlying plots in the image. As will be demonstrated through experiments, the method is highly suitable for delineation of irregularly spaced plots in a field.

The rest of this paper is organized as follows. In section 2 we describe the field trials on which aerial images were taken and present the plot alignment algorithm proposed in this paper. In section 3 we present the results of validation and testing of the algorithm on images of the field trials. We end with a discussion of results and suggestions for future work in section 4 and a statement of conclusions in section 5.

## 2. Materials and Methods

### 2.1. Field Trials

**Figure 2:**
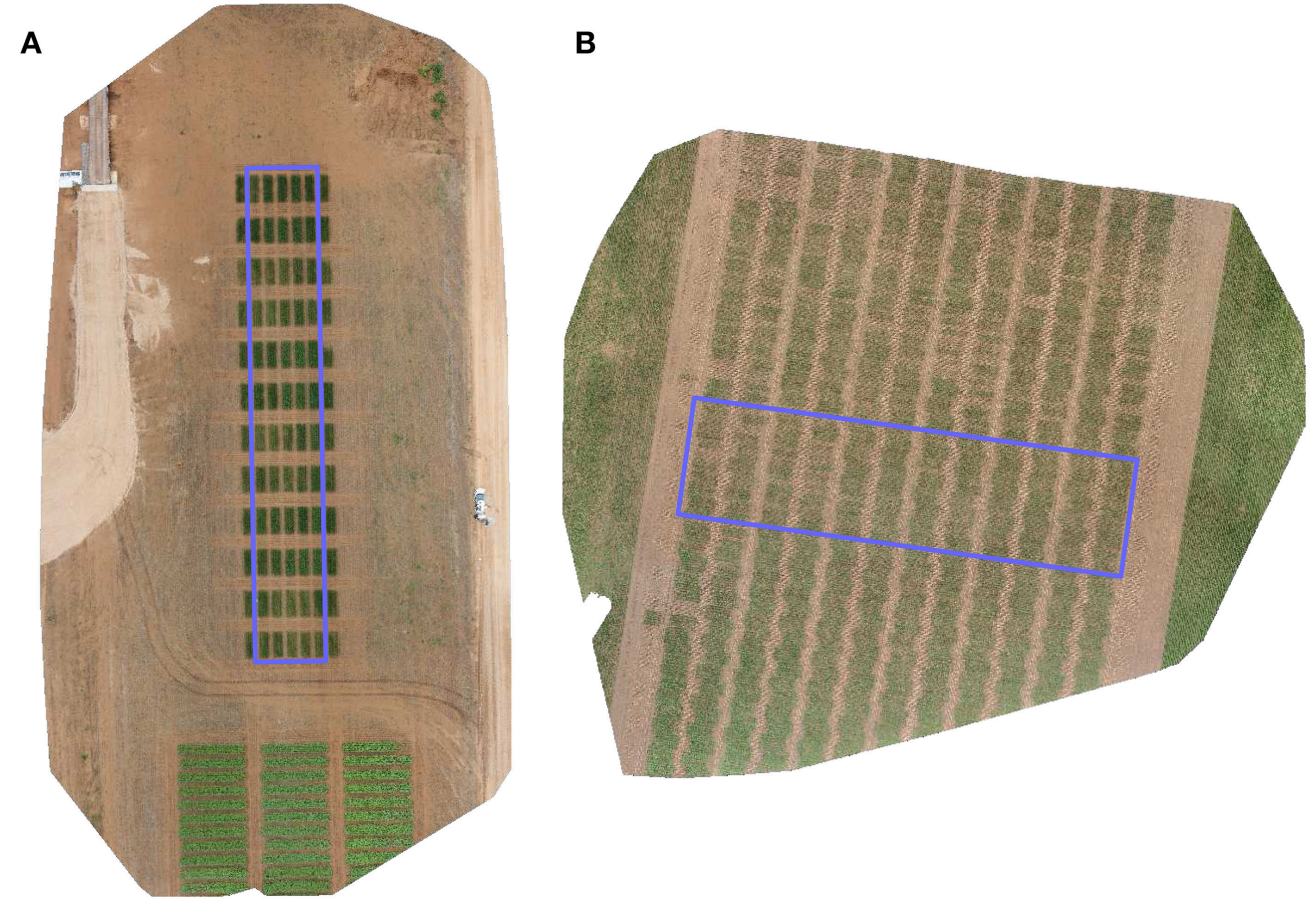
RGB orthomosaic image of the trial sites used for **(A)** validation, and **(B)** testing of the proposed method. Rectangular region signifies an approximate extent of the trials under consideration of this study.

The images used in this exercise were taken of two separate field trials which are shown in Figure 2.

Our validation image is of a trial conducted to observe the differential growth response of wheat to fertilizer treatment. A set of ten contrasting varieties (*Drysdale, Excalibur, Gladius, Gregory, Kukri, Mace, Magenta, RAC875, Scout, Spitfire*) of spring wheat (*Triticum aestivum L*.) in six replicates were laid out in a 5 × 12 randomized split-block design of 60 plots. Additional plots, not included in the trial were added at either end of the rows to attenuate edge effects on the border plots. The nominal plot dimensions were 1.2 × 4 m, comprising 6 rows each with an inter-row spacing of 0.2 m. Three replicates of each variety were selected for treatment, based on a top dressing of 16:8:16 N-P_2_O_5_-K_2_O applied 35 days after sowing and a top dressing of Urea applied 62 days after sowing. The other three replicates of each variety received no fertilizer treatment and served as controls. The experimental site was located in Mallala, South Australia (latitude = –34.457062, longitude = 138.481487). The trial was sown on July 8, 2016 and the aerial images were acquired 73 days after sowing. This field trial was best suited for validation since its plots had distinct placement (Figure 2A) allowing for simulated experiments of plot alignment with a rigorous evaluation of parameters.

Our test image is of a trial targeted at phenotyping of three different crosses of wheat for breeding. For each cross, 80 double haploid (DH) lines were planted unreplicated, along with 24 check varieties of soft and hard wheat, replicated twice. The DH lines of each cross was grown in a block of 4 ranges and check varieties were randomized within the block. Further to increase the disease pressure, a highly susceptible line (Morocco) was repeated regularly in each range. Germplasm entries were planted in a 48 × 12 fully randomized layout of 576 single-row plots of size 0.3 × 5 m each. The experimental site was located between Mallala and Balaklava, South Australia (latitude = 34.301192, longitude = 138.482500). The trial was sown on May 25, 2016 and the aerial images were captured 72 days after sowing. This field trial was ideally suited for testing the alignment algorithm because its plots were inherently misaligned due to variability in sowing position (Figure 2B).

### 2.2. Aerial Image Acquisition and Processing

Aerial images were collected by RX100 MIII Digital Compact camera (Sony Corp., Japan) mounted on a 3DR Solo Quadcopter drone (3D Robotics Inc., USA). Flight mission was planned using ground control station software, Mission Pilot (ArduPilot). The UAV followed a path directed by the controller to cover the geographical extent of a sites. The camera acquired 20.1 megapixel images at 2 second intervals from a constant height of 30 meters, maintaining an image-overlap of more than 80%. Radiometric calibration was performed using a standard reflectance panel (MicaSense Inc., USA) which was photographed before commencement of a flight. The captured images were stored in compressed JPEG format. A photogrammetry software, Pix4Dmapper v4.0 (Pix4D, Switzerland) was employed to process raw aerial images into an orthomosaic image. The orthomosaic images were generated at a resolution of 0.8 cm per pixel and stored in georeferenced TIFF format.

A graphical user interface was developed in MATLAB R2018b (Mathworks Inc., USA) for laying out a grid over the orthomosaic image. The tool enabled interactive placement of a grid of cells over a field image before proceeding with the automatic alignment function, which aligned each cell so as to correspond to an individual plot in the field. The following two dimensional geometric transformations are supported for placement of the grid:

- Translation: Displace the grid position.
- Rotation: Orient the grid at an angle.
- Scaling: Resize the grid to given plot dimensions.
- Shifting: Modify the grid cells to match plot spacing.

**Figure 3:**
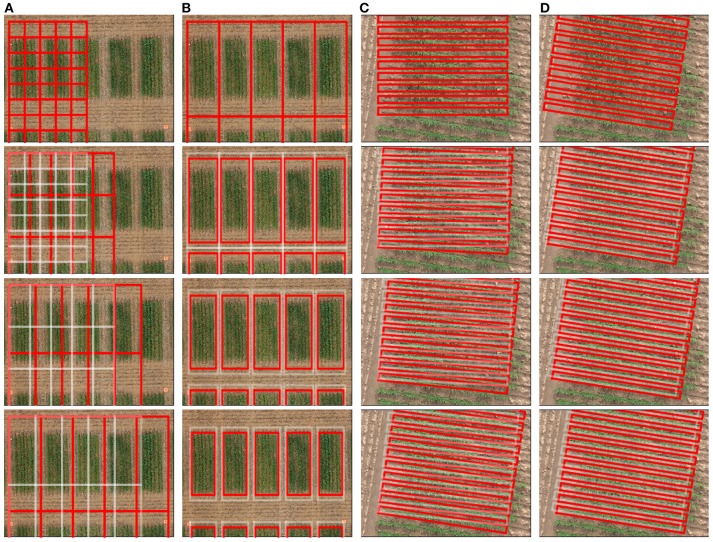
Illustration of interactive grid manipulation functions (top to bottom) offered in the graphical user interface. **(A)** Scale cells to adjust to the plot size **(B)** Shift margin between cells to remove alleyways **(C)** Rotate cells to align with the orientation of trial **(D)** Translate cells to position over the plots.

The specification of a grid may be achieved using the above functions, in any order, combination and as many times as necessary, as exemplified in Figure 3. In addition to the grid layout, a cell sequence corresponding to the research trial can also be specified. The position and attribute(s) of the grid can be exported in the *shapefile* format for use in external software.

### 2.3. Grid Alignment Function

**Figure 4:**
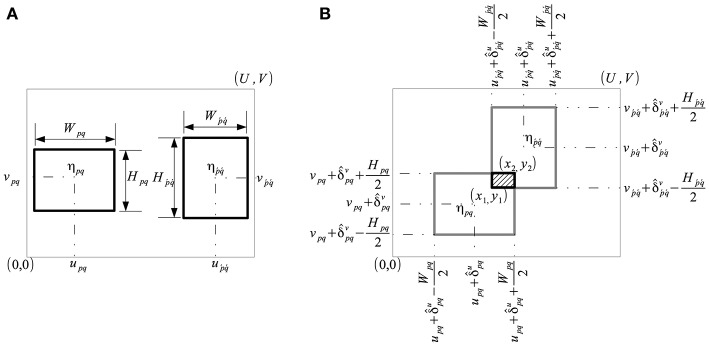
Characterization of grid cells in a discrete scalar field **S** ∈ ℝ^*U*×*V*^. **(A)** A plot is characterized by a cell η_*pq*_ of size *W*_*pq*_×*H*_*pq*_ centered at (*u*_*pq*_, *v*_*pq*_), and its neighbor $\eta_{\overset{´}{p}\overset{´}{q}}$. **(B)** Displacement of cells η_*pq*_ and $\eta_{\overset{´}{p}\overset{´}{q}}$ by parameters $\left({û}_{pq},{\hat{v}}_{pq}\right)$ and $\left({û}_{\overset{´}{p}\overset{´}{q}},{\hat{v}}_{\overset{´}{p}\overset{´}{q}}\right)$ resulted in overlap.

We formulate a cost function to find the optimal alignment of a grid of rectangular cells, where the size, shape and orientation of the cells is fixed, whereas their relative distance varies. Consider a grid $G=\left\{\eta_{pq}\right\}$ of *P* × *Q* cells which represent the plots, where *P* is the number of rows (*P*≥1) and *Q* is the number of columns (*Q*≥1). A cell η_*pq*_ is characterized by its fixed size, *W*_*pq*_ × *H*_*pq*_, corresponding to the width (*W*_*pq*_ > 0) and height (*H*_*pq*_ > 0) of the plot, and its position (*u*_*pq*_, *v*_*pq*_), corresponding to the spatial coordinates of the center of the plot. The grid is located in a finite discrete scalar field **S**:ℝ^*U*×*V*^ → ℝ (as shown in Figure 4A) such as a vegetation index signifying the level of greenness on a numeric scale.

The von Neumann neighborhood of cell η_*pq*_ in the grid can be represented by a set of index pairs corresponding to the cell's immediate neighbors in rows/columns. The index pair $\left(\overset{´}{p},\overset{´}{q}\right)$ of a neighboring cell thus satisfies

(1) $$\begin{array}{l} \left(\overset{´}{p},\overset{´}{q}\right)\in \left\{\left(p-1,q\right),\left(p,q-1\right),\left(p+1,q\right),\left(p,q+1\right)\right\} \end{array}$$

where indices $\overset{´}{p}$ and $\overset{´}{q}$ must satisfy the inequalities $1\leq \overset{´}{p}\leq P$ and $1\leq \overset{´}{q}\leq Q$ to be valid neighbors. Therefore, a cell can be neighbor to 2, 3, or 4 cells depending on its location (corner, side or internal to the grid).

Our objective is to find the optimal displacement ${\hat{\theta }}_{pq}=\left({\hat{\delta }}_{pq}^{u},{\hat{\delta }}_{pq}^{v}\right)$ with which to move a grid cell η_*pq*_ from its initial position at (*u*_*pq*_, *v*_*pq*_) to its optimal position $({\hat{u}}_{pq}=u_{pq}+\delta_{pq}^{u}\;,v_{pq}=v_{pq}+{\hat{\delta }}_{pq}^{v}),$, without change in lateral dimensions. The optimal displacement vector is a member of the discrete set

(2) $$\begin{array}{l} \Theta =\left\{\theta_{pq}=\left(\delta_{pq}^{u},\delta_{pq}^{v}\right):|\delta_{pq}^{u}|\leq \Delta_{pq}^{u}\text{and}|\delta_{pq}^{v}|\leq \Delta_{pq}^{v}\right\} \end{array}$$

where $\Delta_{pq}^{u}$ and $\Delta_{pq}^{v}$ are the respective bounds on the orthogonal components of the 2D vector displacement.

Expressing an arbitrary displacement in a subset of Θ as $\theta_{pq}=\left(\delta_{pq}^{u},\delta_{pq}^{v}\right)=\left(w+{\hat{\delta }}_{pq}^{u},h+{\hat{\delta }}_{pq}^{v}\right)$, we define the intra-cell energy *d*_*pq*_, in the scalar field **S**, as

(3) $$\begin{array}{l} d_{pq}=\sum_{\begin{array}{c} -\frac{W_{pq}}{2}\leq w\leq \frac{W_{pq}}{2}\\ -\frac{H_{pq}}{2}\leq h\leq \frac{H_{pq}}{2} \end{array}}S(w+u_{pq}+{\hat{\delta }}_{pq}^{u},h+v_{pq}+{\hat{\delta }}_{pq}^{v}) \end{array}$$

which is the energy of **S** accumulated within the bounds of the cell η_*pq*_, hence termed as the intra-cell energy. The intra-cell energy signifies the level of vegetation inside a cell at a certain location.

In contrast to the intra-cell energy, consider the mutually shared energy resulting from the interaction of cell η_*pq*_ with its neighbors, $\eta_{\overset{´}{p}\overset{´}{q}}$, as illustrated in Figure 4B. Following an arbitrary displacement of cells η_*pq*_ and $\eta_{\overset{´}{p}\overset{´}{q}}$, the coordinates of their mutually overlapping region are given by

(4) $$\begin{array}{l} (x_{1},y_{1})=(\max\left\{u_{pq}+{\hat{\delta }}_{pq}^{u}-\frac{W_{pq}}{2},u_{\overset{´}{p}\overset{´}{q}}+{\hat{\delta }}_{\overset{´}{p}\overset{´}{q}}^{u}-\frac{W_{\overset{´}{p}\overset{´}{q}}}{2}\right\},\\ \;\max\left\{v_{pq}+{\hat{\delta }}_{pq}^{v}-\frac{H_{pq}}{2},v_{\overset{´}{p}\overset{´}{q}}+{\hat{\delta }}_{\overset{´}{p}\overset{´}{q}}^{v}-\frac{H_{\overset{´}{p}\overset{´}{q}}}{2}\right\}) \end{array}$$

(5) $$\begin{array}{l} (x_{2},y_{2})=(\min\left\{u_{pq}+{\hat{\delta }}_{pq}^{u}+\frac{W_{pq}}{2},u_{\overset{´}{p}\overset{´}{q}}+{\hat{\delta }}_{\overset{´}{p}\overset{´}{q}}^{u}+\frac{W_{\overset{´}{p}\overset{´}{q}}}{2}\right\},\\ \;\min\left\{v_{pq}+{\hat{\delta }}_{pq}^{v}+\frac{H_{pq}}{2},v_{\overset{´}{p}\overset{´}{q}}+{\hat{\delta }}_{\overset{´}{p}\overset{´}{q}}^{v}+\frac{H_{\overset{´}{p}\overset{´}{q}}}{2}\right\}) \end{array}$$

We define the inter-cell energy, *g*_*pq*_, in terms of the mutually shared energy in the scalar field **S**

(6) $$\begin{array}{l} g_{pq}=\sum_{\overset{´}{p}\overset{´}{q}}\frac{d_{\overset{´}{p}\overset{´}{q}}}{d_{pq}+d_{\overset{´}{p}\overset{´}{q}}}\sum_{\begin{array}{c} x_{1}\leq w\leq x_{2}\\ y_{1}\leq h\leq y_{2} \end{array}}S(w,h) \end{array}$$

which is the sum of accumulated energy of **S** due to the overlap of η_*pq*_ and its neighbors $\eta_{\overset{´}{p}\overset{´}{q}}$. The inter-cell energy signifies the level of vegetation in the overlapping region of a cell and its neighbors at their respective locations. Note that the term $\frac{d_{\overset{´}{p}\overset{´}{q}}}{d_{pq}+d_{\overset{´}{p}\overset{´}{q}}}$ is the ratio of the intra-cell energy of neighbor $\eta_{\overset{´}{p}\overset{´}{q}}$ to the sum of their respective intra-cell energies. This coefficient attributes the mutually shared energy to a pair of neighboring cells being proportional to their intra-cell energies. When a cell does not overlap with any of its neighbors (*x*_1_ ≮ *x*_2_, *y*_1_ ≮ *y*_2_) then its inter-cell energy *g*_*pq*_ = 0.

Our objective is to maximize the intra-cell energy to encourage alignment, and to minimize the inter-cell energy to discourage overlap. Therefore, we define the net energy, *f*_*pq*_, of cell η_*pq*_ to be,

(7) $$\begin{array}{l} f_{pq}=\frac{d_{pq}-g_{pq}}{W_{pq}\times H_{pq}} \end{array}$$

which is normalized by the area of the cell. The energy of the grid G is then given by the uniformly averaged net energy of all *PQ* cells and the cost function is taken to be,

(8) $$\begin{array}{l} f=\exp\left(-\frac{1}{PQ}\underset{pq}{\sum }f_{pq}\right) \end{array}$$

where the exponential decay function has been used to ensure numerical stability. The cost function *f* forms the basis for the optimization algorithm.

### 2.4. Optimization Algorithm

**Figure 5:**
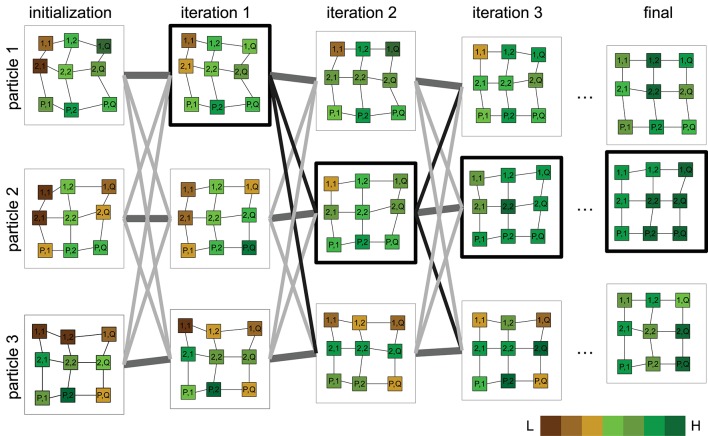
Illustration of the proposed optimization approach for plot alignment. Particles are characterized by a P×Q grid graph where each cell (plot) is initially placed at a uniform random position. A cost function whose output is proportional to underlying vegetation is evaluated for all particles. Particle positions are updated, weighted by the difference between their current and individual best position, best position in the neighborhood and the best overall particle position so far (outlined bold). Particles progressively align with regions of vigorous vegetation. The final alignment is given by the particle with the minimum cost achieved upon termination. (Pseudocolor scale from low:L to high:H vegetation index).

The idea of particle swarm optimization (PSO) was initially proposed in Kennedy and Eberhart (1995) and various improvements to the algorithm and parameters have since been proposed (Pedersen, 2010; Mezura-Montes and Coello, 2011). We leverage this algorithm's ability to seek the displacement θ required to align a grid from a random set of solutions Θ as illustrated in Figure 5. The algorithm begins with a swarm of particles Θ ∈ ℝ^*N*×*K*^ (candidate solutions), all but one randomly initialized within the predefined bounds of a search space Δ^θ^ and one particle initialized as null. The cost function (*f*) is evaluated for all particles and the best function value and its corresponding best particle state are recorded. A particle is updated based on its current state, the difference from its best state, and its difference from the best particle among its neighbors. Particles move in a dynamic neighborhood and are iteratively updated until a convergence criterion is satisfied. The final solution is given by the particle with the best function value in the swarm. A more detailed description of the optimization algorithm can be found in the Supplementary Material.

## 3. Results

The ground truth location of all plots was manually labeled in the orthomosaics to validate and test the performance of the alignment algorithm. Denote the 2D displacement vector of the grid cell η_*pq*_ from its initial position (*u*_*pq*_, *v*_*pq*_) in the uniform grid to the ground truth position by $\left({\bar{\delta }}_{pq}^{u},{\bar{\delta }}_{pq}^{v}\right)$, and the 2D displacement vector of the grid cell η_*pq*_ from its initial position in the uniform grid (*u*_*pq*_, *v*_*pq*_) to the computed position by $\left({\hat{\delta }}_{pq}^{u},{\hat{\delta }}_{pq}^{v}\right)$. The Euclidean distance between the ground truth displacement vector and the computed displacement vector was chosen as the basis for error estimation

(9) $$\begin{array}{l} \text{error}=\sqrt{{\left({\bar{\delta }}_{pq}^{u}-{\hat{\delta }}_{pq}^{u}\right)}^{2}+{\left({\bar{\delta }}_{pq}^{v}-{\hat{\delta }}_{pq}^{v}\right)}^{2}} \end{array}$$

This metric served as the criterion used to measure the overall performance. The alignment errors were computed in physical units for all *PQ* grid cells corresponding to the plots. Box and whisker diagrams were utilized to graphically illustrate the error distributions.

The discrete scalar field **S** in the definitions of intra-cell energy (Equation 3) and inter-cell energy (Equation 6), was defined in terms of information obtainable from the channels of an RGB image. To be precise, we implemented a green-red difference vegetation index defined as,

(10) $$\begin{array}{l} \text{S}\left(u,v\right)=\frac{\text{G}\left(u,v\right)-\text{R}\left(u,v\right)}{\text{G}\left(u,v\right)+\text{R}\left(u,v\right)} \end{array}$$

where **G** and **R** are the values in green and red channel, respectively, at pixel position (*u, v*).

In general any vegetation index which numerically distinguishes plant pixels from the background can be considered as **S**. This point is further elaborated in the Discussion.

### 3.1. Validation

**Figure 6:**
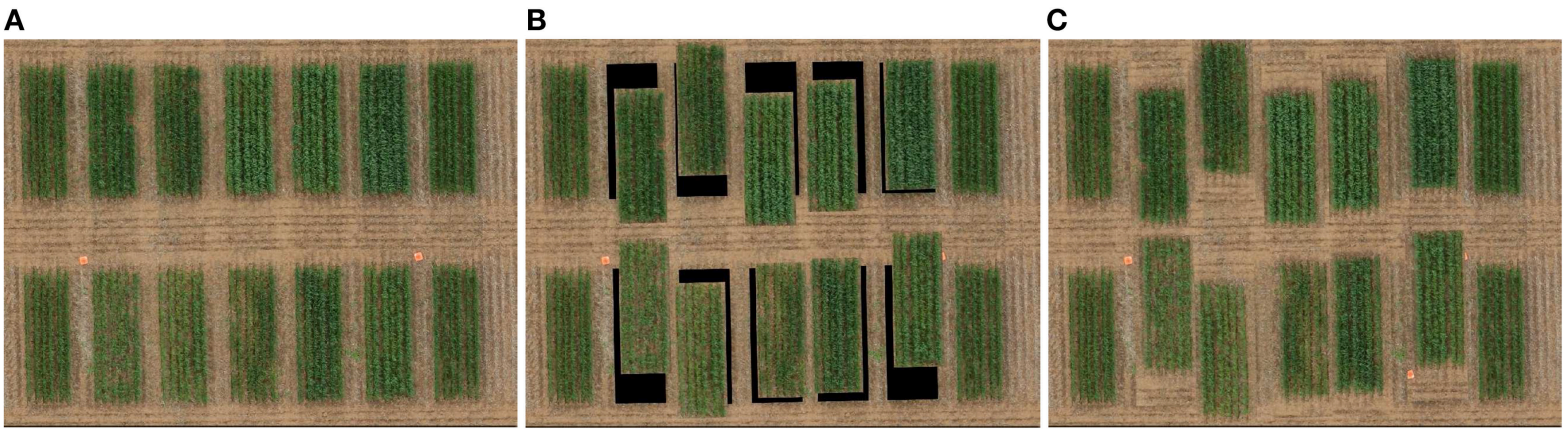
Simulating misaligned plots in an orthomosaic **(A)** Original validation image **(B)** Linear displacement of an image region leaves void areas at the plot's position **(C)** Reflection of an image region displaced from the plot's original position leaves no discontinuity.

From the validation orthomosaic image, we generated new images of artificially distributed plots based on known but random set of displacements. The validation images with known misalignment enabled the calculation of errors and an assessment of the stability of our results. However, simply displacing plots in a mosaic was unhelpful as this created voids in the original position of the plots and thus introduced discontinuities in the image data, as shown in Figure 6B. This problem was addressed by taking a unique approach of reflecting larger, randomly displaced regions around a plot in the mosaic as shown in Figure 6C. The resulting mosaic contained no voids, and the discontinuities were limited to low texture (soil) regions. The modified orthomosaic images with artificially misaligned plots appeared more realistic and were used for validation experiments.

Several aspects pertaining to the initialization, termination, and problem size of the optimization algorithm; as well as the role of cost function were explored using the validation images. These features are discussed in turn below.

#### 3.1.1. Initialization

**Figure 7:**
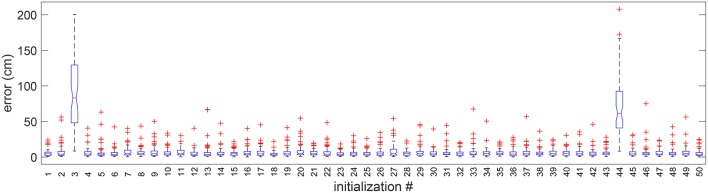
Boxplot of errors for 50 random initializations in 1 random validation trial. Outliers are individually plotted as “+”.

The dependency of the solution on the swarm initialization (see Supplementary Materials) was evaluated by running the algorithm with 50 different swarms. Each swarm is a random set of solutions independently drawn from a uniform distribution. In Figure 7, we present the whisker diagram of errors for each run. It can be observed that 48 initializations resulted in successful alignment, whereas only two resulted in failure. This shows that the optimization algorithm is largely invariant to the initialization.

#### 3.1.2. Random Trials

**Figure 8:**
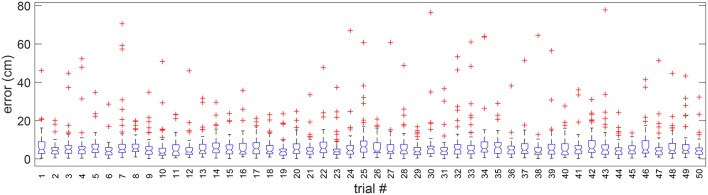
Boxplot of errors in 50 random validation trials. Outliers are individually plotted as “+”.

We tested the adaptability of the algorithm to different randomly simulated trials from the original validation image. In Figure 8, we present the distribution of cell alignment errors for each of the 50 different randomly simulated validation trial images. It can be observed that the median error is 5 cm in general with the existence of a few outliers varying in each trial.

#### 3.1.3. Tolerance

**Figure 9:**
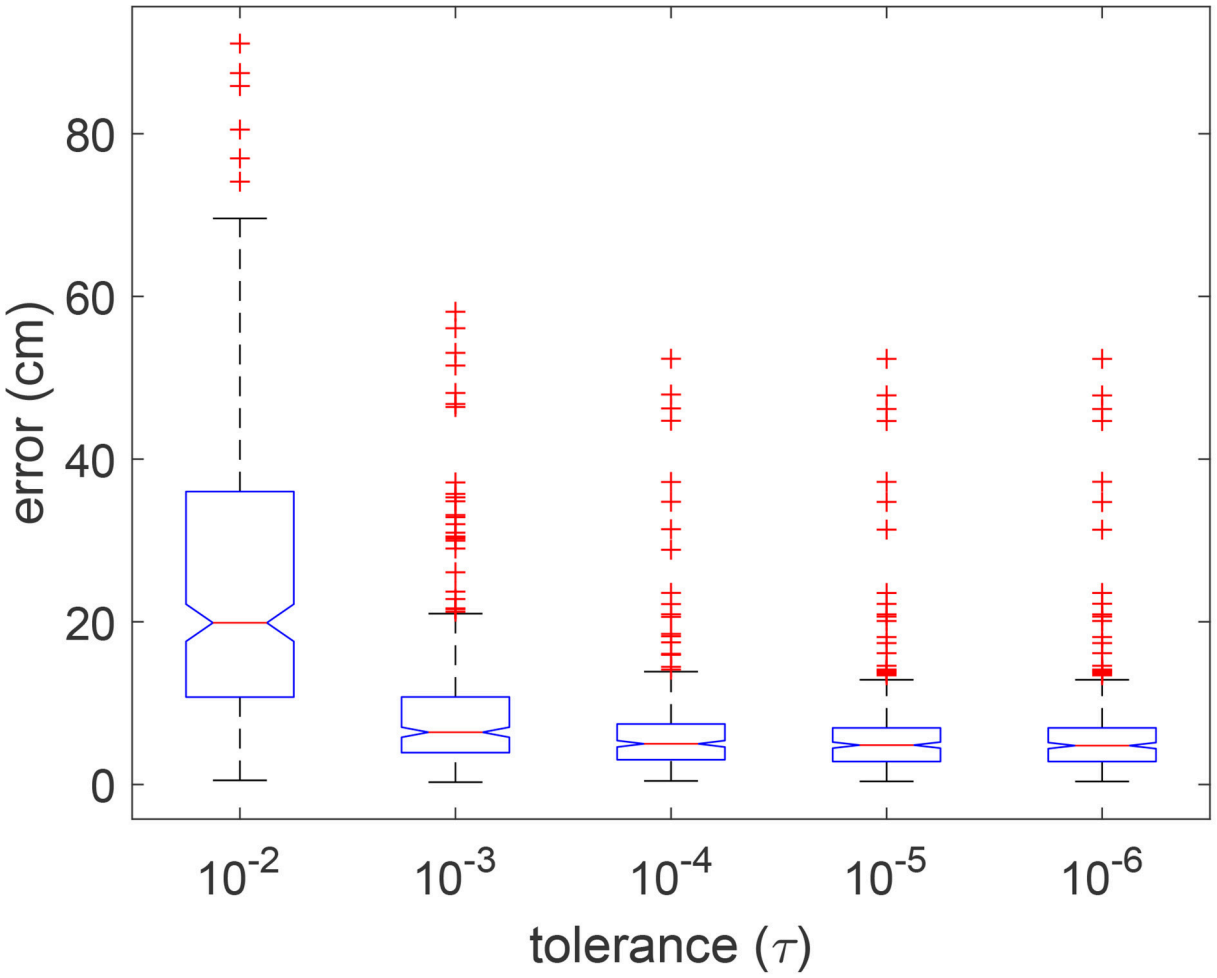
Boxplot of errors for using 5 different tolerance values in 5 random validation trials. Outliers are individually plotted as “+”.

The tolerance value for termination of an optimization procedure can play an important role in the final result as well as for efficiency. Given the nature of the optimization, the minimum cost function value is maintained or improved with each iteration. The procedure is terminated if the change in minimum value does not differ by more than the specified tolerance, for a fixed set of consecutive iterations. In Figure 9, we show the distribution of errors in five random simulated trials for tolerance values ranging over five orders of magnitude, i.e., from 10^−2^ to 10^−6^. It can be observed that there was no significant improvement in errors for tolerance smaller than 10^−4^.

#### 3.1.4. Swarm Density

**Figure 10:**
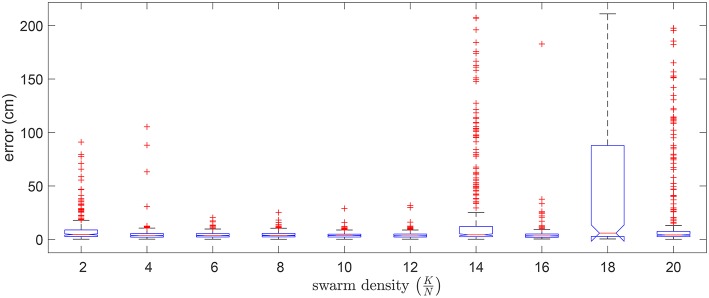
Boxplot of errors for using 10 different swarm sizes in 5 random validation trials. Outliers are individually plotted as “+”.

We also considered how the swarm size affected the quality of the optimized solution. In Figure 10 we show the distribution of errors in 5 random trials for 10 different swarm sizes (*K*) in relation to a fixed problem size (*N*). It can be seen that a swarm density (*K*/*N*) ranging from 6 to 12 generally resulted in lower errors.

#### 3.1.5. Cost Function

We computed the contributions of the two constituent parts of the cost function and evaluated their influence on the overall performance in terms of errors.

The intra-cell energy component of the cost function established a baseline level of performance. The inter-cell energy term penalizes solutions with overlapping cells. To evaluate the usefulness of the inter-cell energy component, we simulated an artificial image with significantly more misalignment in one direction such that alternate pairs of plot rows had no gap. This created a challenging scenario since the absence of a significant gap presents a difficulty in demarcating plot boundary. Furthermore, we paired rows of fertilized plots with those of unfertilized plots. This offered a more challenging scenario because cells tend to overlap or align with plots which have a higher vegetation index due to the greedy nature of intra-cell energy function.

**Figure 11:**
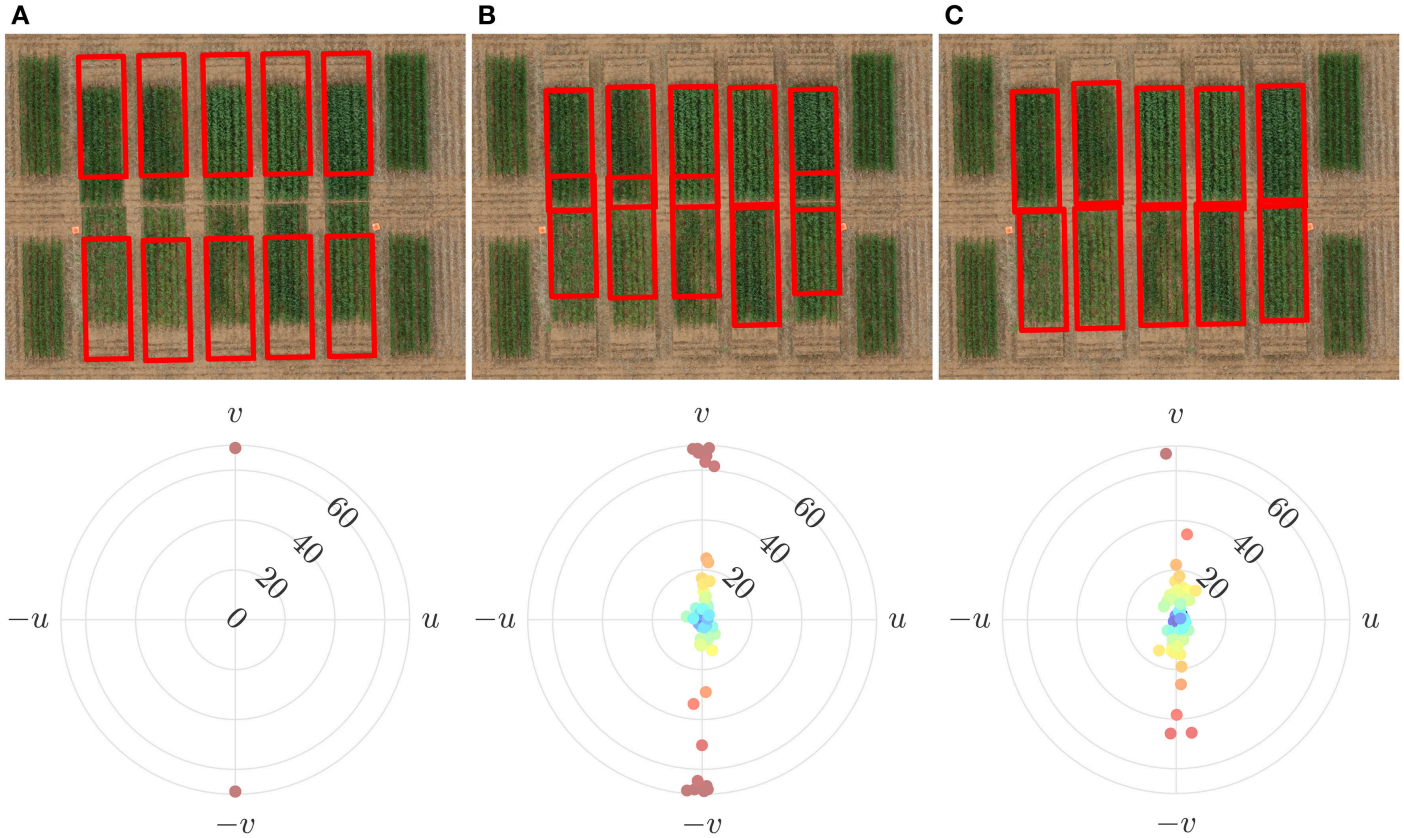
Effect of the constituent parts of the cost function on alignment errors. **(A)** Initially overlaid grid with regular spacing and its alignment error **(B)** Using only the intra-cell energy resulted in cells to overlap with poor alignment **(C)** Using both the intra-cell and inter-cell energy significantly improved alignment.

Figure 11 shows that cell alignment is erroneous when solely based on the intra-cell energy. It has been demonstrated here that alignment of cells of unfertilized plots (which are less vigorous and have low vegetation index values) tends to be biased toward fertilized plots (which are more vigorous and have high vegetation index values). This is as expected since the cost function seeks to align grid cells to areas of high vegetation index. With the addition of an inter-cell energy penalty, the anomaly is significantly reduced. The overlap of nearby cells, is generally limited to non-vegetative regions, i.e., to the gaps between consecutive plots.

### 3.2. Testing

**Figure 12:**
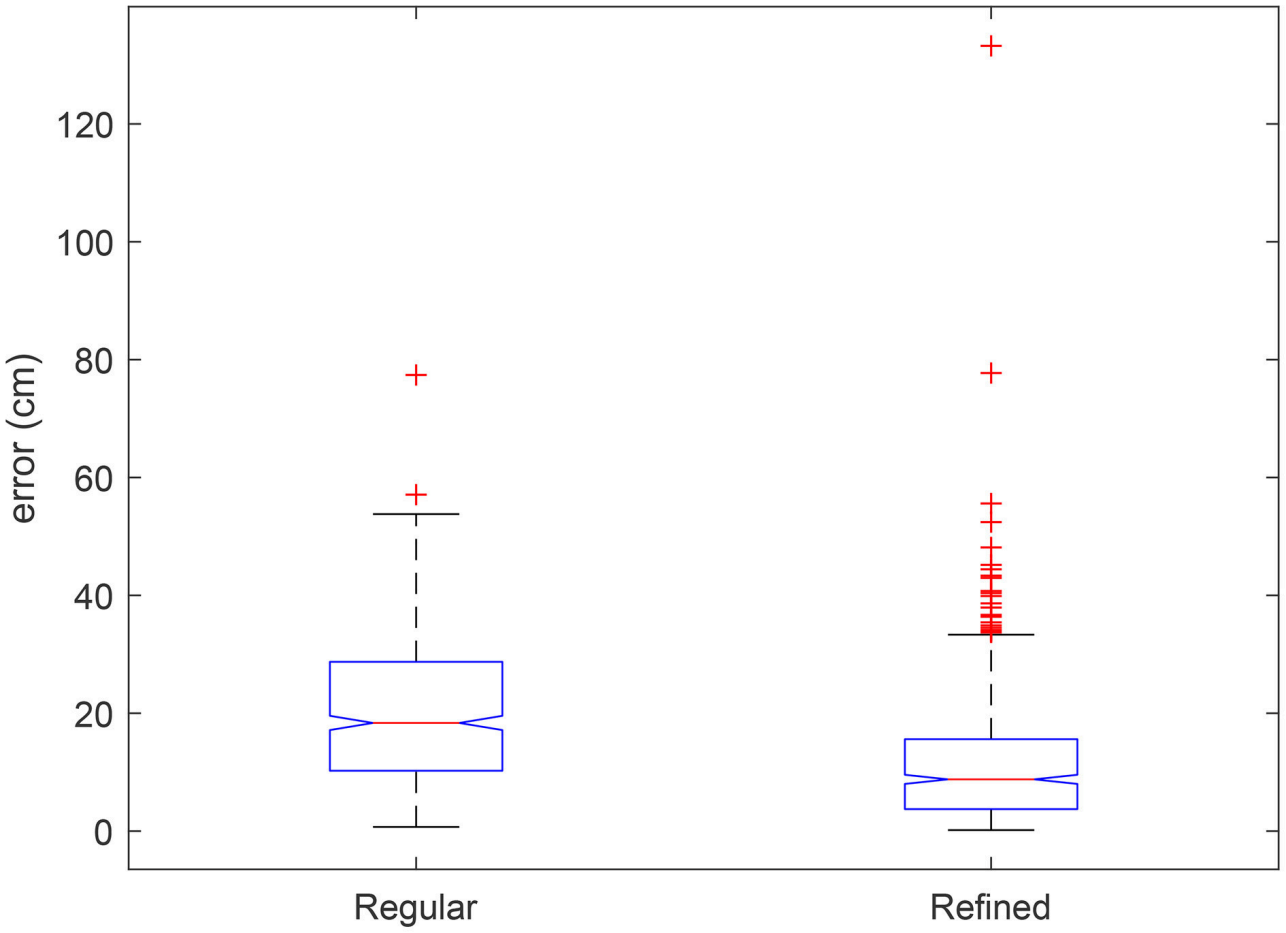
Boxplot of alignment errors of the automatically refined grid compared to errors of a regular grid with uniform spacing on test orthomosaic image. Significant differences are indicated by non-overlapping box notches. Outliers are individually plotted as “+”.

**Figure 13:**
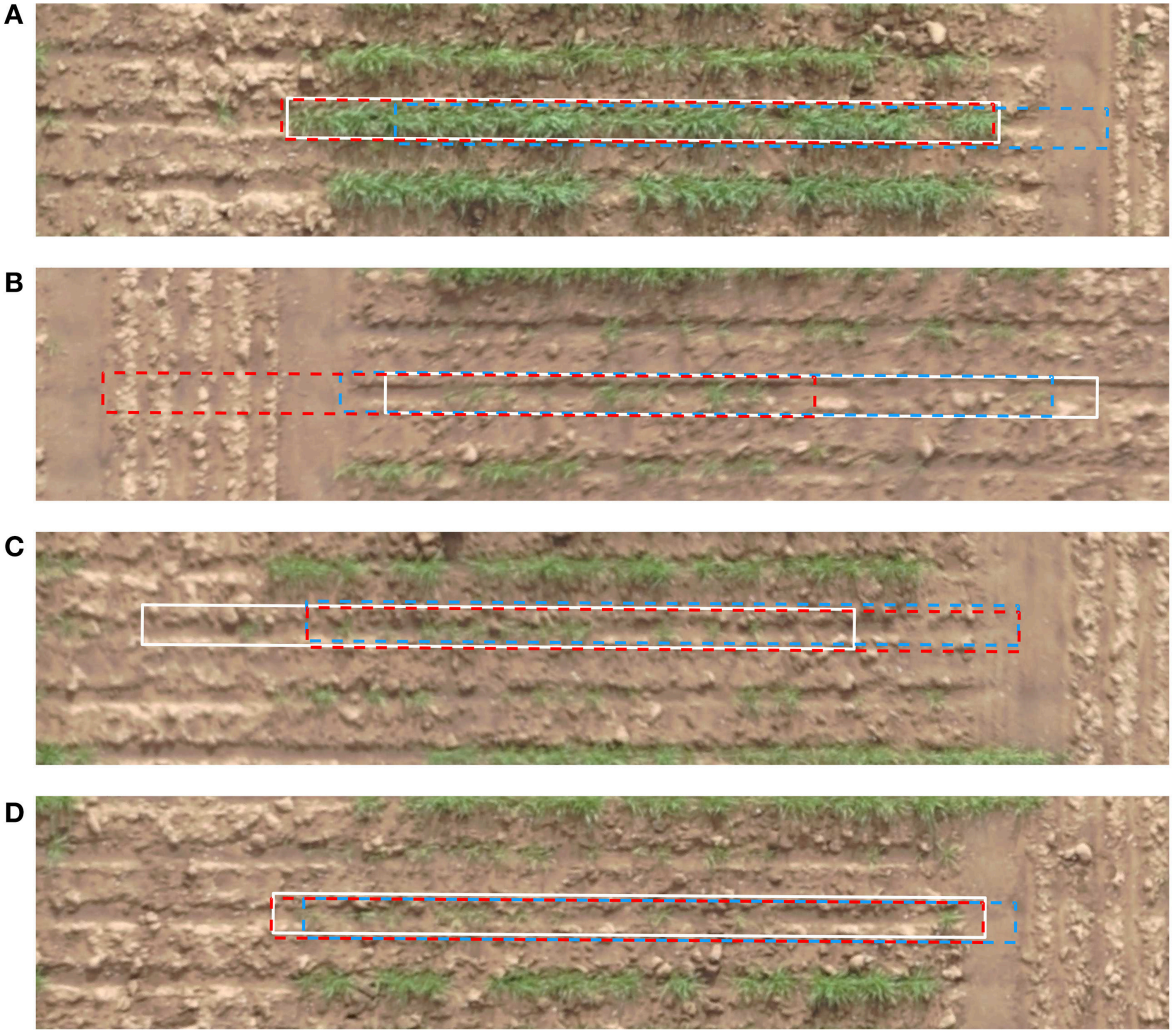
Example of cells aligned by the proposed method (red, dashed) in comparison to the uniformly spaced cells (blue, dashed) and ground truth cells (white, continuous). **(A)** Full emergence, good alignment **(B)** Partial emergence, bad alignment **(C)** Partial emergence, fair alignment **(D)** Partial emergence, good alignment.

The result of cell alignment errors on the test image are summarized in Figure 12. The alignment errors of the automatically refined grid are compared with that of a regular uniformly spaced grid. The cell alignment error is significantly reduced through the application of the proposed algorithm as shown in Figure 12, based on an initialization using the same uniformly spaced grid. To complement these graphical results we visually compare the errors on a few sample plots in Figure 13. The sample plots have been selected to show variety of cases in terms of density of vegetation as well as accuracy of result. It can be seen that in general, the alignment errors are longitudinal, i.e., they lie along the direction of the sowing track. The alignment result is highly accurate in fully emerged plots, one such example from a large majority of these plots is shown in Figure 13A. The alignment can be erroneous in case of partial emergence of seedlings, a rare worst-case scenario of which is shown in Figure 13B. The sparsity of vegetation along the ends of a partially emerged plot can also translate into positional ambiguity as shown in Figure 13C. When seedling growth is present at the extreme edges despite missing in between, the result can still align with the ground truth position as shown in Figure 13D.

#### 3.2.1. Effect on Phenotypic Trait

The potential benefit of accurate plot extraction can be appreciated when it is ultimately used for the analysis of a phenotypic trait. One way to evaluate its advantage is to derive a common trait such as the canopy coverage from extracted plot location. Estimation of canopy coverage requires segmentation of plant pixels from the background which can be accomplished by applying a threshold to the discrete scalar field **S**. Pixels having a higher value than the threshold are regarded as belonging to the plants and vice-versa. We empirically selected a value of threshold to achieve the visually best result by overlaying the segmentation mask over the RGB image. The canopy coverage of plant pixels could then be expressed as a percentage of the total number of pixels in a cell. The coverage was estimated based on a regular grid of uniformly spaced cells, a regular grid of uniformly spaced cells with length trimmed (50%) and an automatically refined grid obtained with the proposed method. The suggested coverage estimates of each grid were compared to the actual coverage values obtained from the ground truth grid.

**Figure 14:**
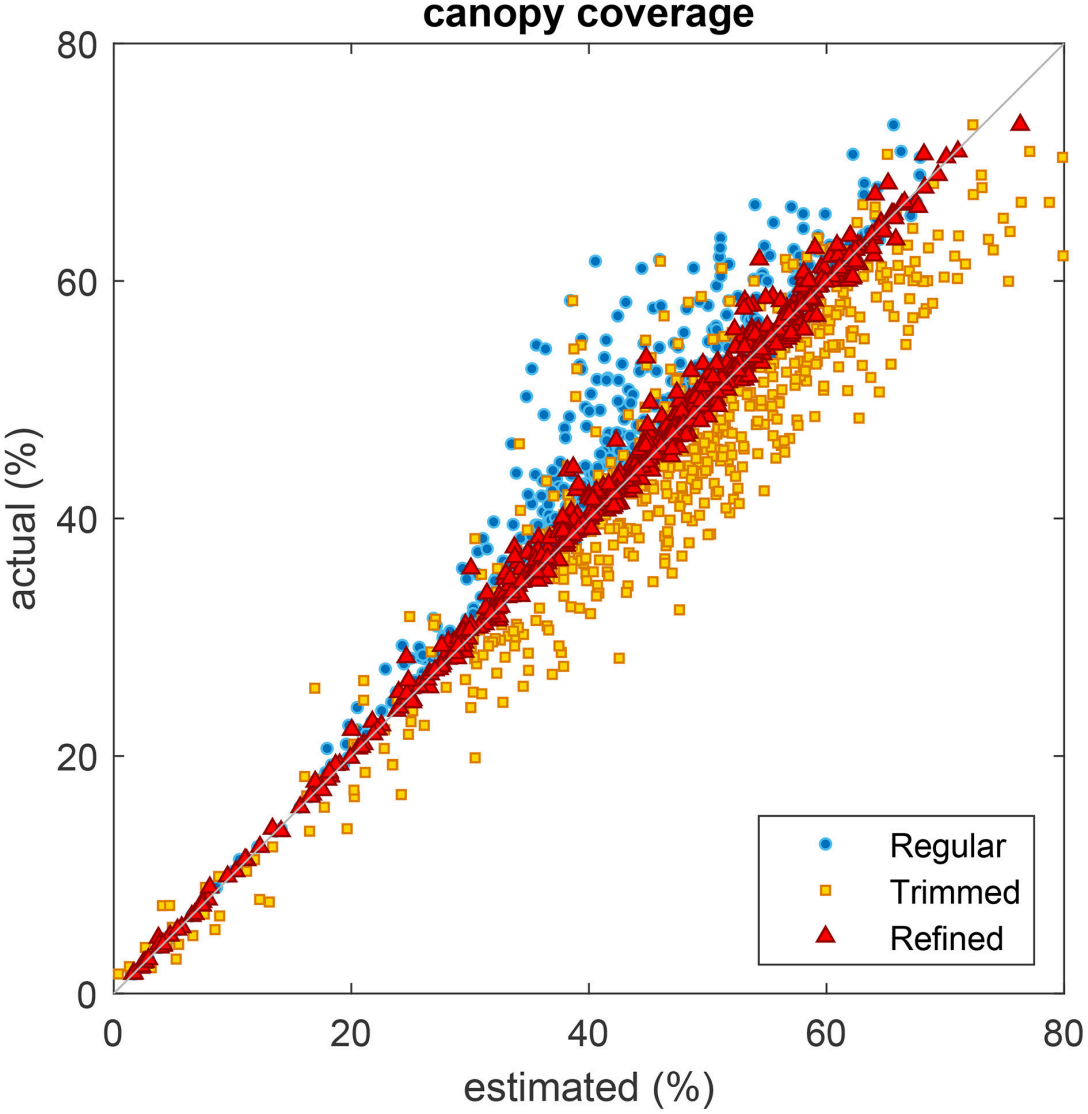
Comparison of plot coverage estimated from three different types of grid with the coverage obtained from the ground truth grid. Regular grid with uniformly spaced cells (blue circles), regular grid with uniformly spaced cells and trimmed edges (yellow squares), and refined grid obtained using the proposed method (red triangles).

Figure 14 shows the scatter chart of canopy coverage obtained using the different types of grid against those of using the ground truth grid. A regular grid underestimated the coverage in most cases as it was not in positional alignment with the plots but rather overlaid on portions of soil. A regular grid with trimmed edge overestimated the coverage as it was generally positioned in the center of plots where vegetation was more likely to be present but did not take the excluded region into consideration. In contrast, a regular grid with automatic refinement returned better coverage estimates corresponding well with the ground truth estimates as its position was correctly aligned.

An analysis of variance (ANOVA) was conducted on plot coverage computed from the different types of grids (see Table 1). The result was significant and a low *p-value* indicated that coverages estimated from regular, trimmed, refined and ground truth grids are not same (*F* = 14.17, *p* = 3.77 × 10^−9^). Further multiple comparison suggested that coverage estimated from either regular or trimmed grid were different from all others, including the ground truth grid. However, coverage estimated from refined grid was different from regular and trimmed grids only.

**Table 1:** Analysis of variance of plot coverage from four different grids (regular, trimmed, refined, and ground truth).

**Source**	**SS**	**dF**	**MS**	**F**	***p***
Groups (between)	0.8519	3	0.28398	14.17	3.77 × 10^−9^
Error (within)	46.0978	2300	0.02004		
Total	46.9498	2303			

*SS, Sum of squares; dF, Degrees of freedom; MS, Mean square; F, F-statistic; p, p-value*.

## 4. Discussion

Any form of image analysis of a remotely sensed field trial, whether it be for assessment of canopy vegetation index (Khan et al., 2018b), estimation of canopy vigor and height (Cai et al., 2018; Khan et al., 2018a), or for estimation of yield by counting heads (Fernandez-Gallego et al., 2018; Hasan et al., 2018; Zhou et al., 2018), must begin with establishing the precise location and perimeter of field plots. Agronomic field trials can have an enormous scope, especially in plant breeding. Consequently, the magnitude of the task to manually demarcate individual plot sites on an image becomes considerable. Assuming a uniform grid of cells based on an expected experimental design (such as those shown in Figure 13) or even further clipping the cells to central portion of plots will almost certainly lead to significant errors in estimates of many phenotypic traits, which are normally quoted per unit area. As it has been demonstrated that canopy coverage, related to green pixel area relative to plot area can be in significant error (underestimated or overestimated) if predicted cell locations are grossly misplaced from the actual plots. Analogous to canopy coverage, vegetation index estimates could be under-represented if soil area was captured instead of true canopy area. Similarly, the assessment of crop germination and yield can be in error as a consequence of the failure to properly align cells with actual plot locations.

The main aim of this paper has been to introduce a robust algorithm to accurately superimpose cells of specified dimensions in an irregular grid over an image of a field trial, so that the positions of grid cells optimally correspond to actual field plot locations. Through the application of a series of validation experiments on simulated images and testing on real image of trial sites, we have demonstrated that the method is accurate, while requiring minimal manual input. Although it can be argued that the extreme displacement of plots (Figure 6) simulated for validation studies is not usually encountered in practice, the accurate results of our algorithm suggest that cell alignment with actual plots in less severe circumstances would be even more easily achieved. The procedure thus offers the potential for automatic application to actual plant breeder trials.

Assuming only fixed dimensions of grid cells, our proposed algorithm, invoking the combination of two complementary (nigh competing) cost functions shows remarkable results, as measured by our error metric in Equation (9). That an intra-cell energy is insufficient to optimize grid position is clear from Figure 11. By not including an inter-cell energy cost function, which penalizes overlap with neighboring cells, can result in a higher energy value when cells overlap green pixel-rich regions of neighboring plots. Our choice of inter-cell function is of course not unique. An extreme alternative inter-cell energy function could operate by simply excluding configurations in which any two cells overlap. In this case

$$\begin{array}{l} g_{pq}=\{\begin{array}{ll} \infty , & x_{1}\leq x_{2},y_{1}\leq y_{2}\\ 0, & \text{otherwise} \end{array} \end{array}$$

which is akin to assuming that cell displacement is “hard limited,” or equivalently that the energy of any overlap is infinite. While this may seem to be a simpler prospect, such a penalization would lead to a high(er) number of rejected moves, thus forcing a greater number of iterations, thereby interrupting the optimization procedure. In contrast, the present approach allows “soft limited” cell displacements, with overlapping cells penalized with a cost proportional to the amount and content of overlap. This results in a more stable and robust optimization.

The proposed energy functions depend on a discrete scalar field which can be derived from the channel(s) of an image. In this study, we used a vegetation index derived from RGB images, which has elsewhere (Khan et al., 2018a,b) been shown to correlate well with normalized difference vegetation index (NDVI). It is, however, also possible to employ a different vegetation index as fundamental determinant, based on e.g., multispectral images. However, our preferred choice was based, at least partly, on the high resolution capabilities of the RGB camera, which can more accurately differentiate plant canopy (foreground) pixels from soil (background) pixels. Arguing on the basis of its correlation with NDVI, the scalar field we used possessed sufficient information about the state of vigor of the canopy.

Finally, from a technical perspective, the proposed cost function is related to the alignment of cells to plots based on an accumulation of underlying vegetation index. While this function has been shown to work in most circumstances, it needs to be recognized that the best objective value does not guarantee the best cell alignment; although they are related, they are not necessarily equivalent. Also, the final optimal solution was found to be largely invariant to the initialization of the swarm. In rare instances where a given initialization fails to lead to a converged solution, a possible corrective measure one can employ is simply to restart/rerun the algorithm with a different random seed initialization.

In future work it could be useful to extend the method to allow for possible variations in cell dimensions in addition to cell positions. However, it is important to reiterate that “total” measures of phenotypic traits such as germination and yield are more appropriately determined by computing the content of a developed canopy within a theoretically sown plot position represented by a fixed size cell. This is one reason why it may be preferable to fix cell dimensions and determine the optimal cell position rather than employ other boundary establishing techniques such as the level set method or the method of active contours (Mumford and Shah, 1989; Chopin et al., 2016), which explicitly capture the actual boundaries of plot canopy but would not be useful for quantifying traits in the same manner.

## 5. Conclusions

The tedious pre-analysis task of identifying field plots in an orthomosaic image of a plant research field trial can be simplified by the automatic registration of plot locations provided by the grid cell optimization algorithm proposed here. Being able to specify the location of separated field plots in a robust and accurate way sets the stage for a subsequent accurate analysis of a range of crop phenotypic traits such as total canopy coverage and canopy vigor. The proposal suits this general purpose and has the potential to be adapted for more specific purposes, such as cell dimension adjustment to capture exact canopy structure.

## Author Contributions

ZK conceived of the project, developed, and implemented the algorithm and prepared the figures of results. SM proposed validation based on simulated images. ZK and SM wrote the paper. Both authors approved the final version.

### Conflict of Interest Statement

The authors declare that the research was conducted in the absence of any commercial or financial relationships that could be construed as a potential conflict of interest.
