# Supplementary material for: An Automatic Field Plot Extraction Method From Aerial Orthomosaic Images
Source: Front Plant Sci. 2019 May 29;10:683. doi: 10.3389/fpls.2019.00683 (PMC6548842; doi:10.3389/fpls.2019.00683)
Supplement: Supplementary file 1 [file Data_Sheet_1.PDF]

frontiers

Supplementary Material: An automatic field plot extraction method from aerial orthomosaic images

1 PARTICLE SWARM OPTIMIZATION ALGORITHM

The Particle Swarm Optimization (PSO) algorithm is inspired by natural collective motion patterns, such as shoaling of fish, flocking of birds and herding of quadrupeds, wherein the movement of an individual is not only governed by its own position but also guided by the position of others in the swarm. Depending on the target(s), the swarm moves and converges to a single or multiple positions. The main benefit of the PSO algorithm is that it does not rely on gradient computation which makes it easily applicable to solving non-convex problems defined by non-differentiable functions. Furthermore, it is largely robust to parameter initialization and is likely to return the same best solution, even in the presence of multiple alternative solutions.

Consider a swarm of K particles in an N-dimensional space characterized by the position $\boldsymbol{\theta} \in \mathbb{R}^{N\times K}$, and velocity, $\dot{\boldsymbol{\theta}} \in \mathbb{R}^{N\times K}$. The particle positions are initialized with random numbers sampled from a uniform distribution within specified bounds

$$\boldsymbol{\theta} = \{\theta_k \in \mathbb{R}^N : |\theta_{k,n}| < \Delta^\theta_n, k = 1,\cdots,K, n = 1,\cdots,N\} \tag{S1}$$

where $\Delta^\theta = \{\Delta^\theta_n \in \mathbb{R}, n = 1,\cdots,N\} \in \mathbb{R}^N$ is a vector of bounds on the domain of particle position. A particle's initial velocity is also randomly initialized within the range of particle position,

$$\dot{\boldsymbol{\theta}} = \{\dot{\theta}_k \in \mathbb{R}^N : |\dot{\theta}_{k,n}| < 2\Delta^\theta_n, k = 1,\cdots,K, n = 1,\cdots,N\} \tag{S2}$$

The current position of a particle is initially taken as the best known position for that particle,

$$\theta^*_k = \theta_k \tag{S3}$$

A cost function (Equation (8)) is evaluated for each particle, $f_k = f(\theta_k)$, and the minimum value over the set of particles in the swarm is

$$f(\hat{\theta}) = \min_{k\in K} f(\theta_k) \tag{S4}$$

such that the corresponding best position within in the swarm is

$$\hat{\theta} = \arg\min_{k\in K} f(\theta_k) \tag{S5}$$

The termination counter c is initialized to 0 and the position of the kth particle is iteratively updated as follows. A random subset of $J = J_{\text{min}} = \max\{1, \lfloor\gamma K\rfloor\}$ particles is formed around the kth particle. Here $0 < \gamma < 1$ is the minimum fraction of particles in its neighborhood. The position of the particle with minimum cost in the neighborhood of the kth particle is assigned to

$$\check{\theta}_k = \min_{j\in J} f(\theta_j) \tag{S6}$$

The particle velocity is updated using a weighted sum of its previous velocity, difference between its current position and best achieved position, and difference between its current position and best particle position in its neighborhood,

$$\dot{\theta}_k = \rho\dot{\theta}_k + \alpha(\theta_k^* - \theta_k) \odot \mu_\alpha + \beta(\check{\theta}_k - \theta_k) \odot \mu_2 \tag{S7}$$

where $\odot$ is the Hadamard (i.e., component-wise) product. The individual adjustment weight (α) and group adjustment weight (β), control the contribution of particles' position difference from individual best and group best position, respectively, to the overall update. The factors $\mu_\alpha \in \mathbb{R}^N$ and $\mu_\beta \in \mathbb{R}^N$ are drawn from uniform random distributions and provide a scaling of the second and third contributory updates to the velocity in the respective dimensions. The inertia factor $\rho \in \mathbb{R}$ together with a particle's previous velocity defines the momentum of that particle in each iteration. The position of the particle is updated using its current position and velocity:

$$\theta_k = \max(\min(\theta_k + \dot{\theta}_k, \Delta^\theta), -\Delta^\theta) \tag{S8}$$

wherein the updated position is truncated at the positional bounds.

If the value of the cost function at the particle's new position is lower than the value at its previous minimum, then the particle's best position is updated with the new position

$$\theta_k^* = \theta_k, \quad \forall f(\theta_k) < f(\theta_k^*) \tag{S9}$$

If the value of the cost function at the particle's new position is also lower than the swarm's minimum then,

$$\hat{\theta} = \theta_k, \quad \iff f(\theta_k) < f(\hat{\theta}) \tag{S10}$$

In case there is no improvement in the minimum cost of the swarm, the size of the particle neighborhood is expanded, $J_{\text{new}} = \min(J_{\text{old}} + J_{\text{min}}, K)$, the termination counter is incremented, $c = c + 1$ and the neighbourhood size is reset $J_{\text{old}} = J_{\text{new}}$. However, if the swarm's minimum cost improves, the particle neighborhood is reset back to the default size $J_{\text{new}} = J_{\text{min}}$ and the termination counter is decremented, $c = \max(0, c - 1)$. Additionally, the inertia is adaptively updated according to a piece-wise linear function of the optimization progress

$$\rho_{\text{new}} = \begin{cases} 2\rho_{\text{old}}, & c \leq 1 \\ \rho_{\text{old}}, & 1 < c < 6 \\ \rho_{\text{old}}/2, & c \geq 6 \end{cases} \tag{S11}$$

with the reset $\rho_{\text{old}} = \rho_{\text{new}}$. The above function initially boosts momentum and then gradually reduces it as the swarm moves towards a minimum position.

The algorithm terminates when the relative change in the swarm's minimum cost $f(\hat{\theta})$ over a number c_{max} of iterations is less than a prescribed tolerance (τ).

1.1 Implementation

A MATLAB implementation of the above optimization algorithm was utilized in the proposed grid cell optimization methodology. The cost function was implemented in a vectorized manner so as to be simultaneously applied to a swarm $f : \mathbb{R}^{N \times K} \to \mathbb{R}^K$, where $N = 2PQ$ is the number of parameters. The default swarm size was $K = \min(100, 10 \times 2N)$ which was initialized with $K - 1$ random particles plus a null particle $\theta_k = 0 \in \mathbb{R}^N$. Equal values of the adjustment factors for individual ($\alpha = 1.5$) and group ($\beta = 1.5$) movement were used with a minimum neighbour fraction of $\gamma = 0.25$. The reproducibility of

the algorithm was ensured by a fixed random seed so that a repeated run with the same seed achieved the same solution.
